# Antipsychotics: 70 Years

**DOI:** 10.3390/biomedicines11113070

**Published:** 2023-11-16

**Authors:** Giovanni Lentini, Serge Mignani

**Affiliations:** 1 Department of Pharmacy-Pharmaceutical Sciences, University of Bari “Aldo Moro”, 70125 Bari, Italy; 2 Centre d’Etudes et de Recherche sur le Médicament de Normandie (CERMN), University of Caen Normandy (UNICAEN), 14032 Caen, France; serge.mignani@staff.uma.pt; 3 CQM—Centro de Química da Madeira, MMRG, Universidade da Madeira, Campus da Penteada, 9020-105 Funchal, Portugal



*To Davide.*

*I am not Theo, but you were close to Vincent in your troubled life.*

*I wonder if you are both shining on starry nights.*



The discovery of the first neuroleptic chlorpromazine is generally considered the Big Bang of modern psychopharmacology [1]. The introduction of related major tranquilizers in the 1950s substantially altered the lives of patients with schizophrenia, and a tremendous reduction in institutionalized patients was registered. Political decisions contributed to deinstitutionalization, but undoubtedly, this **result was facilitated by** the availability of effective medication [2]. The efficacy of these psychotropic agents against fundamental symptoms of schizophrenia facilitated their definition as antipsychotics. Still, they often lead to a low quality of life for patients and expose them to stigma and discrimination [3]. Iatrogenesis often hampers patient compliance and promotes discontinuation. The major psychiatric disorders, including psychosis, schizophrenia, bipolar disorder and Alzheimer’s disease, are still a therapeutic challenge, and better tolerable and more specific treatments need to be developed [4].

We conceived this Special Issue as an opportunity to take stock of the situation, obtain an up-to-date landscape on the most recent developments, and promote advances in the challenging area of the most disabling psychiatric disorders. A total of 24 papers (13 articles and 11 reviews) were published, and they are listed below in chronological ascending order.

As shown in Table 1, the contributors hail from diverse world regions with Europe and Central Asia predominating. The contributions displayed type diversity ranging from real-world studies to historical perspectives. Unfortunately, no article reported on medicinal chemistry endeavors; this disappointing outcome might reflect the attrition in the development of new small molecules as antipsychotics. The repurposing strategy for chemotherapic application of known antipsychotics [5] was the object of no paper herein, and this might be related to the toxicity of currently used antipsychotics [6].

The articles in this collection encompass a diverse set of topics and include historical perspectives, pre-clinical and clinical studies with the latter predominating. 

To fully understand the antipsychotic clinical breakthrough, one should consider what the state of mental illness was in the past. Thus, we suggest the readers start from the two impressive historical reviews proposed herein (contributions 11 and 17). 

The controversial off-label use of antipsychotics in child and adolescent psychiatry was critically evaluated: limited efficacy and safety concern issues were highlighted and pharmacovigilance warnings were evoked (contributions 1, 3 and 6). On the other hand, antipsychotics are commonly used in the elderly where polypharmacy may raise drug–drug interaction concerns (contributions 4 and 5). As for each xenobiotic, the antipsychotic clinical outcome depends on pharmacogenetics, a field that deserves further studies and unified protocols for determining objective antipsychotic response status (contributions 4 and 7). Precision medicine may profit from single-nucleotide polymorphism studies which may indicate patients with treatment-resistant mental disorders (contribution 24). Genotyping may also offer a way to recognize the individuals at risk of developing parkinsonism related to haloperidol treatment (contribution 9). On the other hand, antipsychotics may affect the brain network and the effects on synaptic plasticity and functional connectivity between brain regions might condition both acute and chronic response (contribution 8). Other phenotypical changes involve the hydrolyzing activity of abzymes (i.e., catalytic antibodies) with an influence on immune response (contribution 22).

Social settings have a role to play in determining clinical outcomes during the treatment with antipsychotics, and different therapeutic patterns can be found between different forensic homogeneous populations (contribution 10).

The route of administration and doses **may affect** patient compliance: long-acting injectable antipsychotics and high-dose strategies seem preferable for patients with severe schizophrenia (contribution 13); however, treatment discontinuation is still a major challenge, and individualized treatment is suggested to improve adherence (contribution 18).

A major concern in antipsychotic use arises from possible ECG alteration and polypharmacy generally adds complexity (contribution 12). Other toxicological issues stem from metabolic changes, and metabolic interventions should be part of daily practice when administering antipsychotics (contribution 14). Metabolic issues are particularly relevant to second-generation antipsychotics, including clozapine and related compounds―the so-called pines. For this sub-group of relatively novel antipsychotics, a rapid (one-week) onset of action was indicated in previous studies. Still, the meta-analysis reported herein concludes that data sparsity limits conclusiveness when chronic schizophrenia is concerned, and points to the need for further study (contribution 15). Conversely, atypical antipsychotics are the drugs of choice to treat dextromethorphan-induced psychotic signs due to their better efficacy and safety profile than typical haloperidol in the short-term course (contribution 16). 

Except for clozapine, currently available antipsychotics are effective against positive signs in ~70% of patients [7]. The rate of success against negative symptoms is by far lower. However, serotonin–dopamine activity modulators represent a possible therapy to reduce negative symptoms (contribution 20), and novel agents with diverse non-dopamine D_2_ receptor targets are currently explored [8]. Possible new targets might be the endocannabinoid system (contribution 19) and trace amine-associated receptor 1 (contribution 23). Clozapine is the only answer for patients with treatment-resistant schizophrenia. However, a subgroup of patients, the so-called ultra-treatment-resistant schizophrenia patients, fails to respond. Attempts are being made to improve clozapine efficacy through the use of electroconvulsive therapy (contribution 21). 

All the above topics were analyzed and discussed in this Special Issue shedding light on such a complex matter. The quality of all contributions seemed generally high to us. At the moment when this Editorial was written, the Special Issue cumulatively received more than 40,000 views and 40 citations. We hope that our editorial efforts will meet the expectations of both our Editors and scholars. More importantly, may this Special Issue contribute to the formidable task of finding novel and more efficacious therapeutic answers to severe mental illness by stimulating new ideas and collaborative endeavors.

## Figures and Tables

**Table 1:** Articles published in the Special Issue: An overview.

Paper No.	Focus	Contribution Type	Geographic Representation
1	Off-label use; child and adolescent psychiatry; conduct disorder	Review	East Asia and the Pacific: India
2	Antipsychotics and neurological soft signs in schizophrenia	Article: cross-sectional study	Europe and Central Asia: Romania
3	Off-label use; child and adolescent psychiatry; addictology	Article: disproportionality analysis	Europe and Central Asia: France
4	Pharmacogenetics; drug metabolism; polypharmacology; drug–drug interactions; molecular docking	Article: in vitro and in silico metabolism studies	East Asia and the Pacific: China Europe and Central Asia: Germany
5	Polypharmacy; geriatric psychiatry	Article: real-world data	Europe and Central Asia: Germany
6	Child and adolescent psychiatry; Feeding and eating disorders	Systematic review	Europe and Central Asia: Italy
7	Pharmacogenetics	Review	Europe and Central Asia: Poland
8	Neurobiology of schizophrenia; brain network plasticity; treatment response and resistance	Systematic review	Europe and Central Asia: Italy
9	Gene polymorphism and iatrogenic parkinsonism	Article: genotyping	Europe and Central Asia: Croatia
10	Forensic psychiatry; polypharmacy	Article: real-world data	Europe and Central Asia: Switzerland
11	Delusional disorder treatment	Review: a historical perspective	Europe and Central Asia: Spain United States and Canada: Canada
12	ECG changes; polypharmacy	Article: cross-sectional study	Middle East and North Africa: Jordan
13	Route of administration and doses vs. treatment outcome	Article: real-world data	Europe and Central Asia: Spain
14	Metabolic risk	Article: real-world data	Europe and Central Asia: Italy
15	Multi-acting receptor-targeted antipsychotics; ‘pines’; time to onset of action and time to maximum antipsychotic effect	Systematic review	Europe and Central Asia: Denmark
16	Iatrogenic psychosis; dextromethorphan	Review	Europe and Central Asia: Poland
17	Antipsychotic development	Review: a historical perspective	United States and Canada: USA
18	Therapy discontinuation	Article: real-world data	Europe and Central Asia: Italy
19	New targets; endocannabinoid system; FAAH and MGL inhibitors	Review: medicinal chemistry perspective	Europe and Central Asia: Italy, Portugal and France
20	Negative symptoms; serotonin–dopamine activity modulators	Review	Europe and Central Asia: Italy
21	Treatment-resistant schizophrenia; ultra-treatment-resistant schizophrenia; electroconvulsive therapy	Article: real-world data	Europe and Central Asia: Croatia
22	Immune response; abzymes	Article: real-world data	Europe and Central Asia: Russian Federation
23	New targets; trace amine-associated receptor 1	Review	Europe and Central Asia: Russian Federation
24	Precision medicine; treatment-resistant schizophrenia	Article: real-world data	Europe and Central Asia: Italy and Germany

## References

[1] Boyd-Kimball D., Gonczy K., Lewis B., Mason T., Siliko N., Wolfe J. (2019). Classics in Chemical Neuroscience: Chlorpromazine. ACS Chem. Neurosci..

[2] Siafis S., Davis J.M., Leucht S. (2021). Antipsychotic Drugs: From ‘Major Tranquilizers’ to Neuroscience-Based-Nomenclature. Psychol. Med..

[3] The Lancet (2022). Can We End Stigma and Discrimination in Mental Health?. Lancet.

[4] Taub S., Krivoy A., Whiskey E., Shergill S.S. (2022). New Approaches to Antipsychotic Medication Adherence–Safety, Tolerability and Acceptability. Expert Opin. Drug Saf..

[5] Lin W.-Z., Liu Y.-C., Lee M.-C., Tang C.-T., Wu G.-J., Chang Y.-T., Chu C.-M., Shiau C.-Y. (2022). From GWAS to Drug Screening: Repurposing Antipsychotics for Glioblastoma. J. Transl. Med..

[6] Baker N.C., Ekins S., Williams A.J., Tropsha A. (2018). A Bibliometric Review of Drug Repurposing. Drug Discov. Today.

[7] Nucifora F.C., Woznica E., Lee B.J., Cascella N., Sawa A. (2019). Treatment Resistant Schizophrenia: Clinical, Biological, and Therapeutic Perspectives. Neurobiol. Dis..

[8] Spark D.L., Fornito A., Langmead C.J., Stewart G.D. (2022). Beyond Antipsychotics: A Twenty-First Century Update for Preclinical Development of Schizophrenia Therapeutics. Transl. Psychiatry.

